# Dopamine Grafted Iron-Loaded Waste Silk for Fenton-Like Removal of Toxic Water Pollutants

**DOI:** 10.3390/polym11122037

**Published:** 2019-12-09

**Authors:** Md Shipan Mia, Biaobiao Yan, Xiaowei Zhu, Tieling Xing, Guoqiang Chen

**Affiliations:** National Engineering Laboratory for Modern Silk, Soochow University, Suzhou 215123, China; shipan0143@gmail.com (M.S.M.); yanbiao136@163.com (B.Y.); 18771093295@163.com (X.Z.); chenguojiang@suda.edu.cn (G.C.)

**Keywords:** waste silk, dopamine, iron particles, wastewater treatment

## Abstract

Dispersion of iron was achieved on waste silk fibers (wSF) after grafting of polydopamine (PDA). The catalytic activity of the resulting material (wSF-DA/Fe) was investigated in Fenton-like removal of toxic aromatic dyes (Methylene Blue, Cationic Violet X-5BLN, and Reactive Orange GRN) water. The dye removal yield reached 98%, 99%, and 98% in 10–40 min for Methylene Blue, Cationic Violet X-5BLN, and Reactive Orange GRN, respectively. The catalytic activity was explained in terms of the effects of temperature, dyes, and electrolytes. In addition, the kinetic study showed that the removal of dyes followed pseudo-1st order adsorption kinetics. These findings allow envisaging the preparation of fiber-based catalysts for potential uses in environmental and green chemistry.

## 1. Introduction 

The control of the water pollution is becoming one of the major challenges worldwide. As for extensive industrialization and subsequently the massive discharge of numerous types of organic toxicants, such as dyes, phenols, and nitroaromatic compounds [1,2,3] releasing in water supplies is becoming a serious issue. However, all of the pollutants are receiving tremendous attention from the water researchers, among them the aromatic dyes received one of the highest concerns due to their high toxic nature, chemical stability, and their resistance to conventional treatment methods [1]. Many approaches have been introduced by the several researchers where hazardous aromatic dyes were removed by various processes like biodegradation, chemical oxidation, adsorption, and so on [4,5,6,7,8]. Various heterogeneous catalytic systems with metal oxides or hydroxides like CuO, ZnO, TiO_2,_ and FeOOH as catalysts, [5,9,10,11] as well as advanced oxidation systems have also been introduced all over the decades [12,13,14]. 

Fenton and Fenton-like treatments showed superior pollutant degradation/reduction efficiency compared with microbial oxidative metabolism, and physical adsorption also has some drawbacks [15,16,17,18]. Several new treatment configurations were introduced to optimize the Fenton process including a photo-assisting process to reduce iron supply [19,20], use of solid iron, or avoid modifying the natural pH value of wastewater [21], and so on. However, one of the drawbacks of this system still remained the separation of iron sludge in the solution after the treatment and recycling before discharging the treated wastewater. This is a time consuming and costly procedure [22]. Immobilization of iron particles (Fe) may become an appropriate and permanent remediation technique to solve this problem. However, it is important to immobilize Fe onto a carrier that can be easily separated from the contaminated water solution as well as provide no/less harm on subsequent management. The Fe immobilized inpolyacrylic acid(PAA) [23,24], starch [25], and polyglycol [26] reported in different literature are difficult to recycle once they are used to treat contaminants. To avoid secondary water contamination, immobilization of zero-valent iron (ZVI) onto solid supports, for example, polymeric membranes [27] and activated carbon [28], could be an ideal option. Morshed et al. [29] immobilized and stabilized ZVI nanoparticles on fibrous polyester. Very few literatures have been found on the use of textile fibers or fabrics to immobilize iron particles aiming heterogeneous catalytic application. 

Silk fibers are one of the most discussed fibers derived from natural sources due to mechanical and thermomechanical properties. However, due to their exceptional properties and complex production procedures, raw silk fiber spun out from silkworm cocoons is a luxurious material in many applications. Hence, the material constructed by silk would not be cost effective compared with other plant based natural fibers. On the contrary, during industrial process, lots of waste silk fibers or scrap silk fibers are produced, which are considered as waste and available at extremely low cost. Therefore, the increasing challenge of recycling them is another concerning issue. So utilizing waste silk into a sustainable remediation application can be an ultimate alternative.

Due to strong adhesion property, dopamine, a component of marine mussel gaining tremendous attention for surface modification of different materials [17,18,19], is being extensively used to prepare super-hydrophobic, antimicrobial, UV-blocking, conductive, as well as dye adsorption materials. Our previous study reported use of dopamine to prepare silk fabric with hydrophobicity, flame retardancy, and UV shielding properties [30]. However, no study reported the effectiveness of dopamine grafted waste silk fibers to prepare heterogeneous catalyst for Fenton-like wastewater treatment application. 

In this work, dopamine was grafted on the surface of silk fibers by rapid oxidative polymerization followed by loading/immobilization of Fe particles on PDA grafted waste silk fibers. Hence, a fiber-based catalyst was prepared for heterogeneous Fenton-like removal of toxic aromatic dyes. The surface morphology and chemical composition of resultant catalyst were characterized by scanning electron microscopy (SEM), energy dispersive spectroscopy (EDS), and Fourier transform infrared (FT-IR) spectroscopy. The effectiveness of catalytic property was assessed under the influence of H_2_O_2_ concentration, dye concentration, temperature, and electrolytes.

## 2. Experimental

### 2.1. Materials

Analytical grade dopamine hydrochloride (98.5%), sodium perborate(NaBO_3_·4H_2_O), ferric chloride hexahydrate (FeCl3·6H_2_O), hydrogen peroxide (H_2_O_2_, 33%), and ferrous sulfate heptahydrate (FeSO_4_·7H_2_O) were purchased from Shanghai Lingfeng Chemical Reagent Co., Ltd. (Shanghai, China) and used as received. Waste silk (average fiber diameter 10 μm) were obtained from Nantong Nafuer Clothing Co., Ltd (Nantong, China). All the dyes (Methylene Blue, Cationic Violet X-5BLN and Reactive Orange GRN) were purchased from Tianjin Tianshun Chemical Dyestuff Co., Ltd (Tianjin, China). 

### 2.2. Methods

#### 2.2.1. Preparation of Dopamine Grafted Waste Silk 

**Scheme 1:**
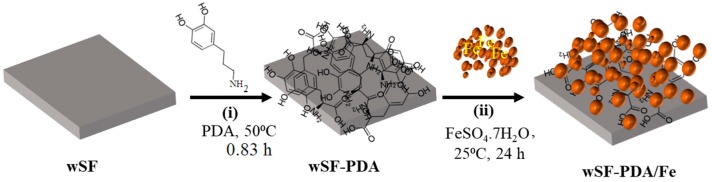
Schematic illustration of the preparation process of (**i**) grafting of polydopamine (PDA) on waste silk fibers (wSF) and (**ii**) loading of iron on dopamine grafted waste silk fibers (wSF-PDA).

Scheme 1 illustrates the pathways used for grafting of dopamine on waste silk. Typically, 1.60 g of waste silk was wetted by deionized water as a pre-treatment. Then, 0.6 g of dopamine hydrochloride and 0.16 g of FeCl_3_·6H_2_O were dispersed in 300 mL deionized water. Wet waste silk fibers were added in dopamine solution and placed in a shaking water bath for 20 min followed by addition of 0.55 g of NaBO_3_·4H_2_O, the final solution was stirred at 50 °C for 50 min. The resultant dopamine grafted waste silk fibers (wSF-PDA) were rinsed and dried overnight in ambient condition.

#### 2.2.2. Preparation of wSF-PDA/Fe

The loading of iron on dopamine grafted waste silk fibers were carried out using chemical adsorption and incorporation method as illustrated in Scheme 1. Here, 15.54 g of FeSO_4_·7H_2_O were dissolved in 1400 mL deionized water in a ceramic tray and stirred until uniform dispersion. Then, the prepared (as Section 2.2.1 described) dopamine-grafted waste silk fibers were immersed in the iron solution and kept at 25 °C for 24 h. The resultant wSF-PDA/Fe undergone successive rinse and stored in a desiccator before analysis and use. 

### 2.3. Material Characterizations 

Fourier transform IR spectroscopy of pristine and iron-loaded wSF was carried out using a Nicolet-5700 Fourier transform infrared spectrometer (MA, USA). The sample to be tested was cut into powder and sampled by potassium bromide. The scanning range was 600–4000 cm^−1^ and the number of scans was 120 times. The samples were drilled before IR analysis, and background spectra were recorded on air. Surface morphologies of all wSF (before and after dopamine grafting and iron loading) were analyzed using desktop scanning electron microscope (Hitachi Ltd., Tokyo, Japan) at an accelerating voltage of 15 kV. Prior to SEM, samples were sprayed with a conducting resin followed by sputter coating with carbon films having a deposition depth of about 10 nm. Energy dispersive spectroscopy (EDS) was carried out using BRUKNER axes EDS analyzer mounted with SEM.

### 2.4. Catalytic Activity of wSF-DA/Fe

To study the catalytic performance of wSF-PDA/Fe, the heterogeneous Fenton-like removal of dyes (methylene blue, cationic violet X-5BLN, and reactive orange GRN) in presence of hydrogen peroxide (H_2_O_2_) was investigated. Typically, 70 mL of dye solution (10–81 mg/L) were treated by using 0.1 mg wSF-PDA/Fe 8 μL (0.05–5 mmol/L) of H_2_O_2_ at a specific temperature (25, 50, or 75 °C). A small amount of solution was taken through a Lab Sphere UV-1000F transmission analyzer (Lab sphere, Inc., North Sutton, VA, USA) to determine the specific absorbance at the characteristic peak of the dye, and the corresponding dye residual rate is calculated as per the Equation (1) [31]:

Removal rate% = (*C*_0_ − *C*)/*C*_0_ × 100 (1)

where *C* is the concentration of the dye during removal at different time interval and *C*_0_ is the initial concentration of the dye. *C*/*C*_0_ was calculated at the maximum absorption wavelength of the dye in visible region. For example, methylene blue at 665 nm, cationic violet X-5BLN at 590 nm, and reactive orange GRN at 480 nm. Control experiments using wSF and wSF-PDA with/or without hydrogen peroxide were conducted to investigate the adsorption and catalytic property of the catalyst.

#### 2.4.1. Effect of H_2_O_2_ Concentration on Removal Performance

The effects of different H_2_O_2_ concentrations on dye degradation were investigated. The reaction conditions were as follows: the reaction temperature was 50 °C, the pH of the reaction was about 7, the concentration of the dye solution was 20 mg/L, and the wSF-PDA/Fe was 0.1 g. The concentration of hydrogen peroxide studied was 0.05, 0.1, 0.5, 1, 3, and 5 mmol/L.

#### 2.4.2. Effect of Pollutant Concentration on Removal Performance

The effects of dye concentration on removal efficiency using wSF-PDA/Fe catalyst were examined. All the experiments were carried out at 50 °C, pH = 7 in a shaking water bath where the initial concentration of H_2_O_2_ reagent was 1 mmol/L, and the dopamine grafted iron-loaded waste silk (wSF-PDA/Fe) was 0.1 g. The dye concentrations were studied at 10, 20, 40, 60, and 80 mg/L, respectively.

#### 2.4.3. Effect of Reaction Temperature on Pollutant Removal Performance

The pollutant removal performances of wSF-PDA/Fe as function of different reaction temperatures were studied. Standard dye (20 mg/L) and H_2_O_2_ (1 mmol/L) concentration was used for 0.1 mg of wSF-PDA/Fe catalyst. Three-reaction temperatures (25, 50, and 75 °C) were studied.

#### 2.4.4. Effect of Different Electrolytes (NaCl, Na_2_SO_4_) of Pollutant Removal Performance

In industrial production, dyeing wastewater contains not only dyes, but also many other inorganic and organic additives. Therefore, the effects of different electrolytes (NaCl, Na_2_SO_4_) on the removal efficiency of wSF-PDA/Fe were also investigated. All the experiments were carried out at 50 °C, pH = 7 in a shaking water bath for the 20 mg/L dye using 1 mmol/L H_2_O_2_, and 0.1 g wSF-PDA/Fe catalyst.

## 3. Results and Discussion

The results were presented and discussed in two separate parts, where the first part focused on analysis of waste silk fibers before and after PDA incorporation and iron loading, and the second part conferred the catalytic behavior of prepared Fe loaded waste silk fibers towards removal of various dyes. 

Part 1: Analysis of Waste Silk Fibers Before and After Dopamine Incorporation and Iron Loading

Morphological analysis using SEM, as well as elemental and functional group analysis by means of EDS X-ray and infrared spectroscopy was used to characterize waste silk samples before and after PDA grafting and iron loading immobilization.

### 3.1. Morphological Analysis 

**Figure 1:**
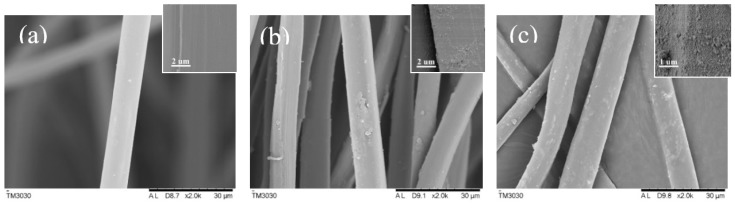
Morphology of waste silk after different treatments: (**a**) wSF; (**b**) wSF-PDA; (**c**) wSF-PDA/Fe.

**Scheme 2:**
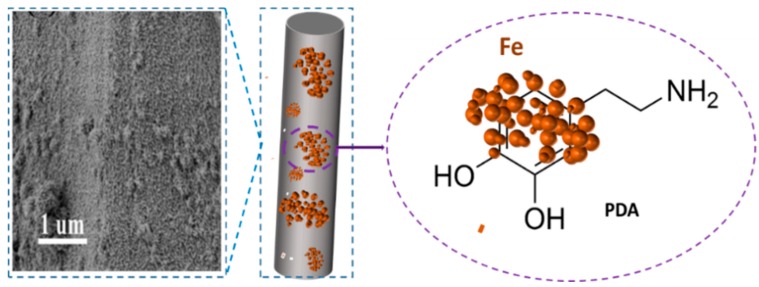
Schematic illustration of iron loading on waste silk.

The changes in surface morphology of the waste silk after different treatment were investigated (see Figure 1). The untreated waste silk (Figure 1a) exhibits a network of a randomly overlapping fibers with a smooth surface. However, a uniform layer of cluster can be observed in PDA grafted waste silk (see Figure 1b), indicating that the PDA is successfully grafted onto the fibers surface through rapid oxidative polymerization. A large amount of particles are found successfully loaded on the surface of wSF-PDA and formed wSF-PDA/Fe. In the case of Fe loaded waste silk fibers (see Figure 1c), several patches appear as regularly shaped clusters [30,32] in the form of layers as demonstrated in Scheme 2. The presence of functional groups on treated waste silk has been further established by EDS X-ray and infrared analysis (see proceeding sections).

### 3.2. Fourier Transform Infrared (FT-IR) Analysis 

**Figure 2:**
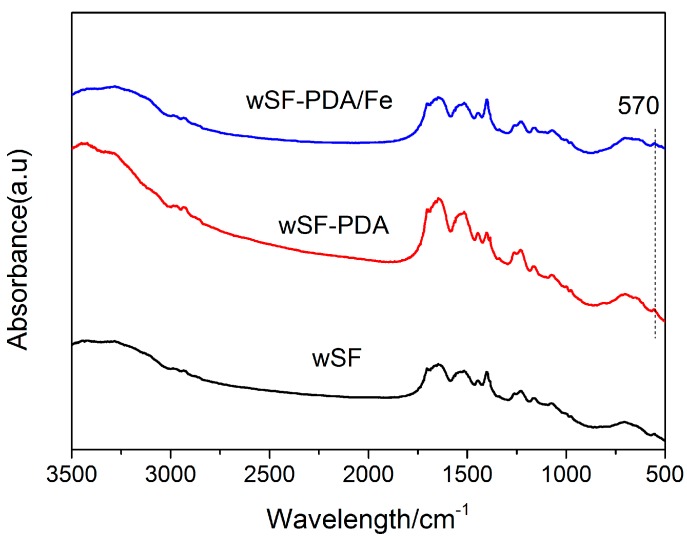
Infrared spectrum of untreated and functionalized waste silk.

In order to further investigate the changes in the structure of the waste silk surface after various surface modifications, the infrared spectrum analysis was carried out as shown in Figure 2. The untreated and treated waste silk demonstrated rough bands in the region 1660–1630 cm^−1^, which were attributed to the stretching vibration of amide I. Other characteristics peaks found at 1545–1525 cm^-1^ are the amide II and 1265–1235 cm^−1^ is the amide III [33,34]. The FT-IR spectra of sample wSF-PDA and wSF-PDA/Fe showed a characteristic bending at 570 cm^−1^ [35,36], indicating the formation of Fe–O due to the presence of PDA [30] and subsequent loading of iron particles.

### 3.3. Loading Analysis

**Figure 3:**
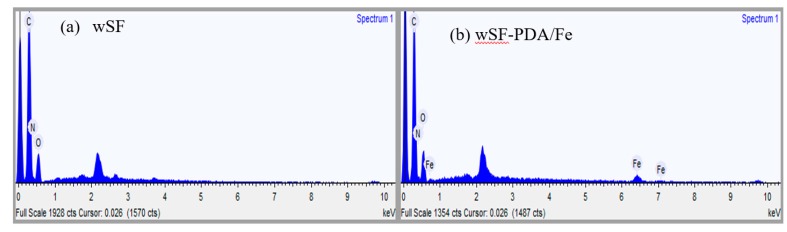
Energy dispersive spectra of (**a**) wSF and (**b**) wSF-PDA/Fe.

**Figure 4:**
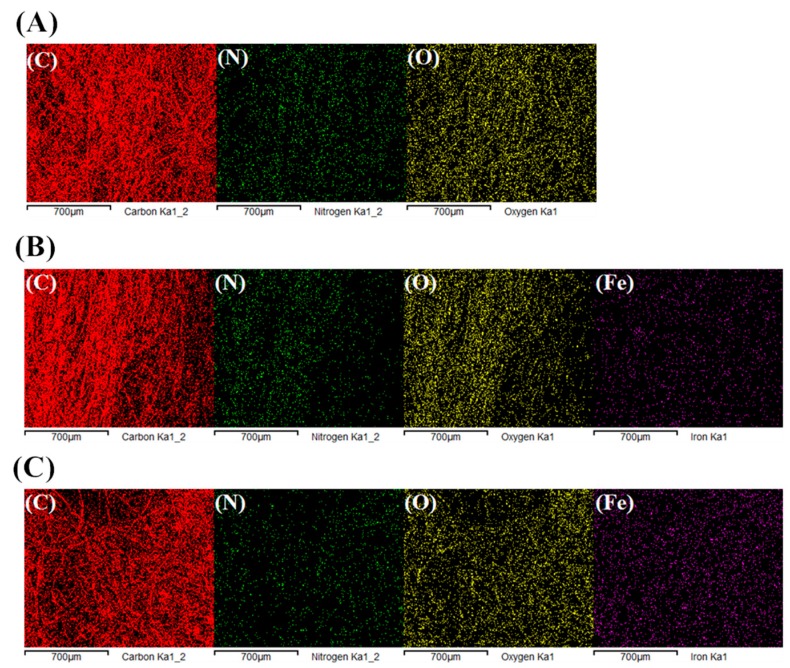
Energy Dispersive X-ray Spectroscopy (**A**) wS, (**B**)wSF-PDA, and (**C**) wSF-PDA/Fe.

EDS X-ray analysis further confirmed the changes in the chemical composition of the fabric surface before and after treatment (see Figure 3 and Figure 4). The surface elemental composition of untreated and functionalized silk fibers is presented in Table 1. It can be concluded that the surface element ratio of N/C of the treated fabric is significantly smaller than the untreated sample [30], indicating successful grafting of PDA. After loading of iron, the peak intensity of Fe element (4.23%) can be noticed in wSF-PDA/Fe. A small quantity of iron element was noticed in wSF-PDA derived from the presence of some Fe-oxides in PDA. 

Part 2: Analysis of Catalytic Fenton-Like Removal Toxic Water Pollutants

Three different dyes (methylene blue, cationic violet X-5BLN, and reactive orange GRN) were adopted to analyze the catalytic Fenton-like property of prepared fibrous catalyst (wSF-PDA/Fe). Dye removal as a function of time and influence of several factors, such as concentration of pollutants, reactants, temperature, and presence of electrolytes, has been investigated by means of UV-Vis colorimetric analysis. 

### 3.4. Degradation of Dyes as a Function of Time

**Figure 5:**
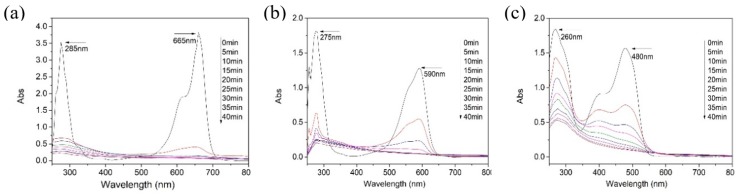
UV-visible absorption spectrum of (**a**) methylene blue, (**b**) cationic violet X-5BLN, and (**c**) reactive orange GRN removal as a function of time. (wSF-PDA/Fe 0.1 g, dye concentration 20 mg/L, H_2_O_2_ concentration 1.0 mmol/L, T = 50 °C, pH = 7).

In order to investigate catalytic behavior of prepared iron loaded waste silk fibrous catalyst, Fenton-like degradation of methylene blue, cationic violet X-5BLN, and reactive orange GRN dye as a function of time was studied and analyzed by UV-Vis spectrophotometer (see Figure 5). Fenton reaction involves the combined use of ferrous ions and hydrogen peroxide (H_2_O_2_) to produce advanced oxidation potential active oxygen species capable of degrading organic contaminants. The dyes selected in this study are common industrial dyes containing an aromatic ring, which are resistant to traditional chemical and biological removal methods. As shown in Figure 5, the characteristic absorption peak intensity of methylene blue at 285 and 665 nm [35] decreased with the function of the reaction time. Once the reaction reached 10 min, the color almost completely disappeared, hence most degradation was achieved by 40 min of catalytic oxidation. Similar phenomena were found in the other two (cationic violet X-5BLN and reactive orange GRN) dyes, where characteristic absorption peak intensity of cationic violet X-5BLN at 275 and 590 nm and reactive orange GRN at 260 and 480 nm disappeared in 20 and 40 min, respectively. Iron loaded wSF fibrous catalyst showed good color removal efficiency, but no visible color removal was observed in control experiments performed using wSF-PDA or wSF-PDA/Fe without one or more reactants (Fe or H_2_O_2_) necessary to initiate Fenton reaction. Nonetheless, trivial reduction in color concentration was observed in various sample (wSF-PDA, wSF-PDA + H_2_O_2_, and wSF-PDA/Fe), which can be due to the adsorption characteristics of heterogeneous catalysts consistent with the literature [29,37,38].

#### 3.4.1. Kinetics of Dye Degradation 

The [instant/initial] absorbance ratio of the methylene blue, cationic violet X-5BLN, and reactive orange GRN dye at λ = 665, 590, and 480 nm (*A*_t_/*A*_0_) respectively, which accounts for the corresponding concentration ratio (*C*/*C*_0_) and allows plotting of ln(*C*/*C*_0_) as a function of time. Model validation of the pseudo-first-order kinetics for color removal with the catalyst is obtained by the linear evolution in time of ln(*C*/*C*_0_), as supported by *R*^2^ values 0.98, 0.99, and 0.98, respectively (see Table 2). Plots summarized in Table 2 show that dye degradation exhibited good linear relationships of ln(*C*/*C*_0_) versus reaction time up to a certain time where maximum number of dyes was degraded following pseudo-first-order kinetics. These results are consistent with those found in previous reports [10,24,33,39,40,41,42].

#### 3.4.2. Postulated Mechanism of Dye Removal

**Scheme 3:**
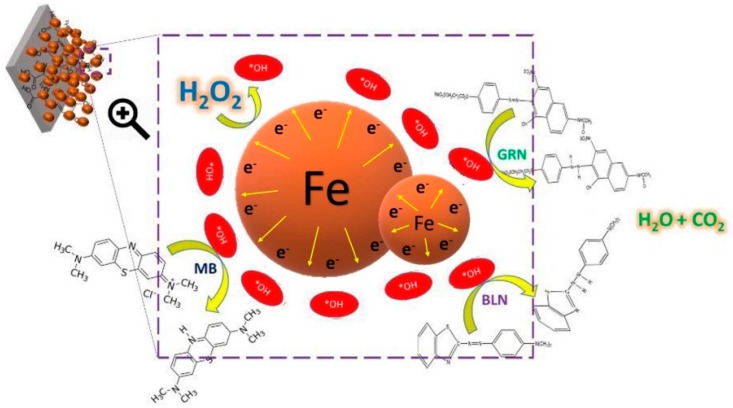
Schematic postulated main mechanism of degradation of methylene blue, cationic violet X-5BLN, and reactive orange GRN dyes.

Based on the results explained in the above sections, a plausible mechanism for removal of all three dyes has been postulated. The underlying mechanisms of removal of methylene blue, cationic violet X-5BLN, and reactive orange GRN dyes in the system of iron loaded PDA grafted waste silk fibrous catalyst were considered for Fenton-like reaction in presence of hydrogen peroxide. The removal of all dyes was attributed to the synergistic effect caused by free radicals and other reactive species formed though a heterogeneous Fenton reaction [29]. The produced free radicals oxidize the dyes into colorless nontoxic substances as illustrated in Scheme 3.

The postulated main reaction mechanism involves three steps as follows:

(i) The process of producing reactive species
  Fe^2+^ + H_2_O_2_→Fe^3+^ + HO^−^ + •OH (1)
(ii) The process of color removal of dyes
  Dyes + •OH→Reaction intermediates(2)
(iii) The process of degradation
  Reaction intermediates + •OH→CO_2_ + H_2_O (3)

### 3.5. Factors Influencing the Removal of Dyes

#### 3.5.1. Effect of Different Samples

**Figure 6:**
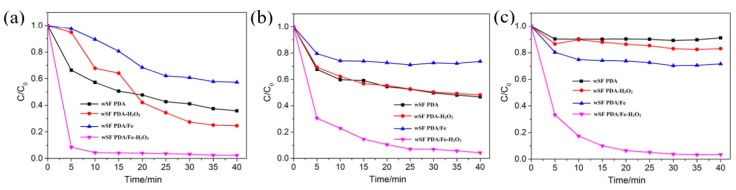
The *C*/*C*_0_ of different samples in the three dye solutions (**a**) methylene blue, (**b**) cationic violet X-5BLN, and (**c**) active orange GRN. (wSF-PDA/Fe 0.1 g, dye concentration 20 mg/L, H_2_O_2_ concentration 1.0 mmol/L, T = 50 °C, pH = 7).

It can be seen from Figure 6 that different samples have a great influence on the degradation dye, and wSF-PDA/Fe-H_2_O_2_ is the most effective for degrading dyes. The addition of wSF-PDA mainly brought about the adsorption of dyes by silk fibers and dopamine dominantly, and the dye removal rate could not meet the requirements. The addition of wSF-PDA and H_2_O_2_ resulted in a lower degradation rate due to the weaker oxidative decomposition of H_2_O_2_ itself and the weak adsorption of wSF-PDA. The wSF-PDA/Fe also played a dominant role in adsorption, and the dye removal rate was not significant. The addition wSF-PDA/Fe and H_2_O_2_ generated a strong reaction system, which effectively increased the release rate of hydroxyl radicals and degraded most of the dyes in a short time.

In addition, it is interesting that wSF-PDA and wSF-PDA-H_2_O_2_ have better degradation for methylene blue and cationic violet X-5BLN. This may be due to the negative charge on the surface of the wSF-PDA sample, which can better absorb cationic dyes and achieve effective degradation.

#### 3.5.2. Effect of H_2_O_2_ Concentration

**Figure 7:**
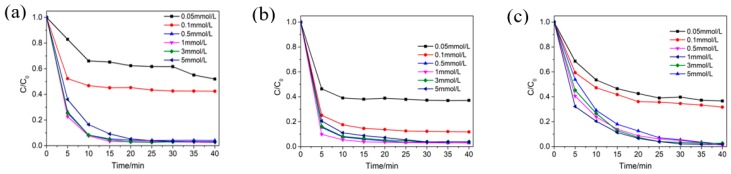
Evolution in time of *C*/*C*_0_ of (**a**) methylene blue, (**b**) cationic violet X-5BLN, and (**c**) reactive orange GRN removal as a function of H_2_O_2_ concentration. (wSF-PDA/Fe 0.1 g, dye concentration 20 mg/L, T = 50 °C, pH = 7).

The effect of H_2_O_2_ concentration (0.05, 0.1, 0.5, 1, 3, and 5 mmol/L) on the removal of dyes was studied. The evaluation of color removal in terms *C*/*C*_0_ as shown in Figure 7 shows that, H_2_O_2_ concentration below 0.5 mmol/L showed poor or insufficient dye removal. However, 0.5 to 5 mmol/L of H_2_O_2_ concentration showed most removal of dyes in similar experimental condition. When the concentration of H_2_O_2_ was 0.05 and 0.1 mmol/L, the dye degradation rates after reaction for 40 min were 48.1% and 57.5%, respectively. At this point, it can be clearly seen that the low concentration of H_2_O_2_ was too diluted to react with the mineralized iron to form sufficient hydroxyl radicals to fully degrade methylene blue. When the concentration of H_2_O_2_ was gradually increased to 0.5, 1, 3, and 5 mmol/L, the final degradation rate reached nearly 97% to 98%, but the efficiency of the reaction was also quite different. The H_2_O_2_ concentration of 1 mmol/L was the appropriate, and the degradation rate was the faster and saturated within 15–20 min, while the reaction rate of high concentration H_2_O_2_ was decreased. This phenomenon indicated that high concentration of H_2_O_2_ cannot increase the degradation rate as linear regression rather showed no improvement after saturation. The similar phenomena can be noticed for other two dyes (cationic violet X-5BLN and reactive orange GRN) as well (see Figure 7b,c).

#### 3.5.3. Effect of Dye Concentration

**Figure 8:**
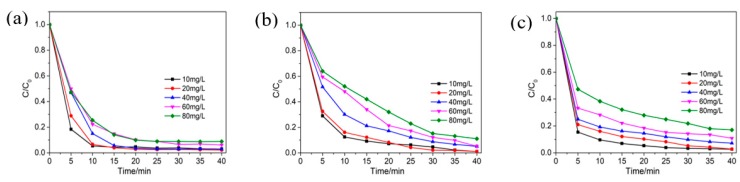
Evolution in time of *C*/*C*_0_ of (**a**) methylene blue, (**b**) cationic violet X-5BLN, and (**c**) reactive orange GRN removal as a function of dye concentration. (wSF-PDA/Fe 0.1 g, H_2_O_2_ concentration 1.0 mmol/L, T = 50 °C, pH = 7).

The concentration of dyes is a precarious parameter of color removal rate, which influences the effectiveness of the removal process. The effect of dye concentration on the removal of dyes were studied in terms of different concentration (10, 20, 40, 60, and 80 mg/L) of dye solutions. Results presented in Figure 8 show a certain influence on the removal rate of all three dyes due to the variation in concentration of the dye solution. The results show (see Figure 8) the most removal of dyes at the initial concentrations from 10–20 mg/L, which moderates upon increase in dye concentration for a specific reaction time. However, for the maximum concentration (80 mg/L) of dye solution, the removal percentage remains above 80%. The highest 90% of removal rate on the maximum concentration are recorded for methylene blue. This may be due to the fact that in the case of high dye concentration, PDA grafted iron loaded waste silk catalyst forms a stable reaction system with H_2_O_2_, at which time the hydroxyl radical is not easily deactivated, and the probability of contact with the dye increases, eventually resulting in a high removal.

#### 3.5.4. Effect of Reaction Temperature

**Figure 9:**
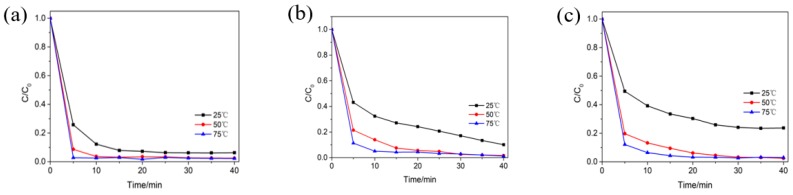
Evolution in time of *C*/*C*_0_ of (**a**) methylene blue, (**b**) cationic violet X-5BLN, and (**c**) reactive orange GRN removal as a function of reaction temperature. (wSF-PDA/Fe was 0.1 g, dye concentration 20 mg/L, H_2_O_2_ concentrations 1.0 mmol/L, pH = 7).

Temperature in a catalytic reaction might increase or decrease the reaction rate, thus the effect of reaction temperature (25, 50, and 75 °C) on the removal of methylene blue, cationic violet X-5BLN, and reactive orange GRN dyes were studied and the results are shown in Figure 9. It can be seen from Figure 9a–c that the reaction temperature has a significant influence on the removal of dyes. In the range of 25–75 °C, the dye removal rate increases with increasing temperature. A slight increase in removal rate can be found in methylene blue removal (see Figure 9a). Whereas, a significant increase has been noticed in cationic violet X-5BLN and reactive orange GRN dyes. Figure 9c shows that, when the reaction temperature is 25 °C, the final dye removal rate of active orange GRN is 77.2% after 40 min reaction; when the reaction temperature is 50 and 75 °C, the final dye removal rate of active orange GRN is about 98.5% after 40 min reaction. However, it is clear that the reaction rate at 75 °C is significantly faster than 50 °C. This phenomenon indicates that the reaction rate under high temperature conditions accelerates the decomposition of H_2_O_2_ into hydroxyl radicals, which increases the velocity of dye degradation reaction. Similar phenomena are found in removal of cationic violet X-5BLN (see Figure 9b).

#### 3.5.5. Effect of Different Electrolytes

**Figure 10:**
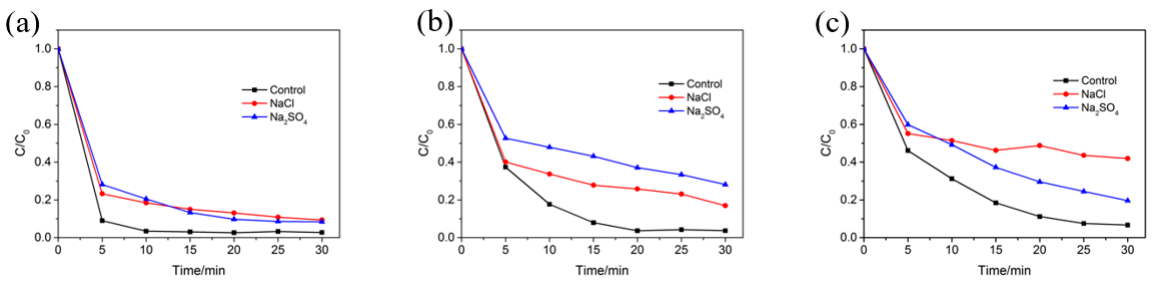
Evolution in time of *C*/*C*_0_ of (**a**) methylene blue, (**b**) cationic violet X-5BLN, and (**c**) reactive orange GRN removal as a function of electrolyte. (wSF-PDA/Fe 0.1 g, dye concentration 20 mg/L, H_2_O_2_ concentration 1.0 mmol/L, T = 50 °C, NaCl, Na_2_SO_4_ concentration 40 g/L, pH = 7).

The traditional dye house uses significant amounts of electrolytes during textile processing, so that considerable amounts of electrolytes are present in wastewater. Consequently, the effect of different electrolytes (NaCl, Na_2_SO_4_) on effectiveness of removal of dyes were studied. Stimulating phenomena are noticed in the results as shown in Figure 10. It can be seen that the electrolyte does influence the removal of degradation of methylene blue in a limited way. For cationic violet X-5BLN and reactive orange GRN, the effect of electrolyte is significant. This may be due to the fact that methylene blue belongs to the class of easily degradable dyes, and the structure of cationic violet X-5BLN and reactive orange GRN are more complicated. Therefore, the electrolyte has a greater influence on the latter two dyes. For different electrolytes, Cl^−^ and SO_4_^2−^ can capture and destroy •OH [43,44], affecting the degradation process of dyes. In general, wSF-PDA/Fe still plays an important role in the degradation of dyes.

## 4. Conclusions

In this report, the loading of iron particle on dopamine grafted waste silk fibers were achieved successfully. The resultant material was investigated by means of physicochemical and catalytic property. SEM and EDS analysis confirmed the loading of PDA and iron particles on the surface of waste silk fibers, which revealed the potentiality of the prepared material as a catalyst in possible Fenton-like removal of aromatic dyes. The results of investigation of H_2_O_2_ concentration, dye concentration, temperature, and electrolytes on dye removal indicated the prepared catalyst showed significant 98%–99% removal dyes in 10–40 min depending on the concentration of H_2_O_2_ used. Postulate mechanism showed the most degradation of pollutants into nontoxic substance. Thus, this study can provide rational foundation towards further advancement in fiber based catalyst for Fenton-like removal of toxic pollutants in water.

## Figures and Tables

**Table 1:** Surface elemental composition and loading analysis of samples (energy dispersive spectroscopy (EDS) analysis).

Samples	Atomic Percentage of Surface Elements			
	C (%)	N (%)	O (%)	Fe (%)
wSF	71.07	10.99	17.94	0
wSF-PDA	76.14	5.56	17.13	1.17
wSF-PDA/Fe	74.47	0.12	21.18	4.23

**Table 2:** Pseudo First-order kinetics for the Fenton-like removal of methylene blue, cationic violet X-5BLN, and reactive orange GRN dye.

Samples	Methylene Blue			Cationic Violet X-5BLN			Reactive Orange GRN		
	^a^*Time* (min)	^b^*k* (min^−1^)	^c^ *R* ^2^	^a^*Time* (min)	^b^*k* (min^−1^)	^c^ *R* ^2^	^a^*Time* (min)	^b^*k* (min^−1^)	^c^ *R* ^2^
wSF-PDA/Fe	10	0.576	0.982	25	0.157	0.997	40	0.065	0.984

^a^Reaction time required for color removal; ^b^*k*: rate constant for the 1st order kinetics and is expressed in min^−1^; ^c^*R*^2^: correlation coefficient of the linear regression.
